# Metabolites in the Dance: Deciphering Gut-Microbiota-Mediated Metabolic Reprogramming of the Breast Tumor Microenvironment

**DOI:** 10.3390/cancers16244132

**Published:** 2024-12-11

**Authors:** Afaf Altrawy, Maye M. Khalifa, Asmaa Abdelmaksoud, Yomna Khaled, Zeinab M. Saleh, Hager Sobhy, Shaimaa Abdel-Ghany, Amany Alqosaibi, Afnan Al-Muhanna, Jawaher Almulhim, Ahmed El-Hashash, Hussein Sabit, Borros Arneth

**Affiliations:** 1 Department of Medical Biotechnology, College of Biotechnology, Misr University for Science and Technology, Giza P. O. Box 77, Egypt; afaf.altrawy@must.edu.eg (A.A.); maye.khalifa@must.edu.eg (M.M.K.); hager.khalil@must.edu.eg (H.S.); hussein.sabit@must.edu.eg (H.S.); 2 Department of Pharmaceutical Biotechnology, College of Biotechnology, Misr University for Science and Technology, Giza P. O. Box 77, Egypt; asmaa.abdelmaksoud@must.edu.eg; 3 Department of Bioinformatics and Functional Genomics, College of Biotechnology, Misr University for Science and Technology, Giza P. O. Box 77, Egypt; yomna.khaled@must.edu.eg; 4 Department of Agriculture Biotechnology, College of Biotechnology, Misr University for Science and Technology, Giza P. O. Box 77, Egypt; zeinab.saleh@must.edu.eg; 5 Department of Environmental Biotechnology, College of Biotechnology, Misr University for Science and Technology, Giza P. O. Box 77, Egypt; shaimaa.ibraheem@must.edu.eg; 6 Department of Biology, College of Science, Imam Abdulrahman bin Faisal University, P.O. Box 1982, Dammam 31441, Saudi Arabia; amgosaibi@iau.edu.sa; 7 King Fahad Hospital of the University, Alkhobar, Imam Abdulrahman Bin Faisal University, Dammam 31441, Saudi Arabia; amuhanna@uod.ed.sa; 8 Department of Biological Sciences, King Faisal University, Alahsa 31982, Saudi Arabia; jalmulhim@kfu.edu.sa; 9 Department of Biomedicine, Texas A&M University, College Station, TX 77840, USA; ahashash@exchange.tamu.edu; 10 Institute of Laboratory Medicine and Pathobiochemistry, Molecular Diagnostics, Hospital of the Universities of Giessen and Marburg (UKGM), Philipps University Marburg, Baldinger Str., 35043 Marburg, Germany; 11 Institute of Laboratory Medicine and Pathobiochemistry, Molecular Diagnostics, Hospital of the Universities of Giessen and Marburg (UKGM), Justus Liebig University, Feulgen Str., 35392 Giessen, Germany

**Keywords:** breast cancer, gut microbiota, tumor microenvironment, metabolic reprogramming, chemotherapy resistance

## Abstract

Breast cancer (BC), a major cause of death among women worldwide, has traditionally been linked to genetic and environmental factors. However, emerging research highlights the gut microbiome’s significant role in shaping BC development, progression, and treatment outcomes. This review explores the intricate relationship between the gut microbiota and the breast tumor microenvironment, emphasizing how these microbes influence immune responses, inflammation, and metabolic pathways. Certain bacterial species in the gut either contribute to or hinder BC progression by producing metabolites that affect hormone metabolism, immune system pathways, and cellular signaling. An imbalance in gut bacteria, known as dysbiosis, has been associated with a heightened risk of BC, with metabolites like short-chain fatty acids (SCFAs) and enzymes such as β-glucuronidase playing key roles in this process. Additionally, the gut microbiota can impact the effectiveness of chemotherapy, as certain bacteria can degrade drugs like gemcitabine and irinotecan, leading to reduced treatment efficacy. Understanding the complex interactions between gut bacteria and BC may pave the way for innovative treatment approaches, including personalized microbiome-targeted therapies, such as probiotics and fecal microbiota transplants, offering new hope for more effective prevention, diagnosis, and treatment of BC.

## Simple Summary

Breast Cancer (BC) is an important disease causing death of many women worldwide. Here the relationship between gastroenteral microbionta and metabolism in the context of BC is investigated and described in detail. The interrelation between BC, metabolite abnormalities and reprogramming, and micronenvironment is described. All information about the various mechanisms by which these bacterial residents may influence disease initiation, progression, and treatment response is collected and summarized. We will see that gut-based biomarkers, and synergy between conventional therapies and microbiome interventions, together will soon be able to open-up new gates for breast cancer therapy.

## 1. Introduction

Breast cancer, the most common malignancy among women globally, exhibits multifaceted complexity in its initiation, progression, and response to therapy [1,2]. While genetic and environmental factors have long been implicated, recent years have unveiled a surprising player in this intricate dance: the gut microbiome [3,4]. This ecosystem of trillions of microbes dwelling within our gastrointestinal tract is no longer considered a passive bystander but rather an active participant in shaping human health and disease.

Intriguingly, evidence is mounting for a bidirectional “gut–tumor axis” specifically influencing breast cancer [5,6,7,8]. This bidirectional dance between gut microbes and cancer cells holds immense potential for understanding and revolutionizing cancer treatment. Therefore, understanding the gut–tumor axis opens doors for novel therapeutic strategies. The gut microbiota (GMB) influences the tumor microenvironment (TME) by orchestrating chronic inflammation, a well-established accomplice in cancer progression [8,9]. Specific bacterial species, like *Fusobacterium nucleatum*, release pro-inflammatory cytokines and chemokines, creating a fertile ground for tumor growth and angiogenesis [10,11]. In contrast, other GMB, such as *Faecalibacterium prausnitzii*, play a counterbalancing role. They promote anti-tumor T cell activity and suppress immunosuppressive cells, fostering an immune response capable of controlling cancer [12].

Recent groundbreaking discoveries have discussed these intricate interactions. Thus, examining the gut microbiome as a novel approach to breast cancer treatment offers immense promise. From personalized prevention to microbiome-based interventions and TME manipulation, the possibilities are vast. While challenges remain, ongoing research holds the potential to reshape the picture of breast cancer treatment, offering safer, more effective, and personalized options for millions of patients.

In this review, beyond mechanistic intricacies, we will critically examine the clinical implications of this gut–tumor connection. We will also analyze how gut microbiota composition might influence breast cancer risk, diagnosis, and response to conventional therapies. Exploring the burgeoning field of microbiota-based interventions, we will discuss the exciting potential of prebiotics, probiotics, and fecal microbiota transplantation in breast cancer prevention, treatment, and even personalized medicine.

## 2. The Gut–Breast Axis

Microbial cells and human host cells work in a complicated symbiotic system within the human body. Our bodies contain a hundred trillion microorganisms that are dispersed throughout the body, including the gastrointestinal tract. These microbes harbor massive colonies of themselves, and their genomes, compared to the host cell genome, are 150 times greater in number [13]. Therefore, they comprise the human body’s second genome and play a crucial role in both normal and disease conditions [14].

Around 300 to 500 different bacterial species can be found in the gut of healthy humans. These species are classified into eight main phyla: *Firmicutes*, *Actinobacteria*, *Proteobacteria*, *Bacteroidetes*, *Verrucomicrobia*, *Lentisphaerae*, *Fusobacteria*, and *Tenericutes* [15,16]. The breast microbiota may originate from the intestine, mammary glands, skin, and breast milk through lactation [17].

Breast tumors differ from normal tissue of the mammary gland in that they have their unique microbiome [18]. Therefore, the gut–breast axis is a new field of research that highlights the relationship between breast health and gut microbiota. Several studies have demonstrated that the gut microbiota plays a critical role in supporting overall health and has a significant impact on many chronic diseases, such as BC [19].

According to a recent study, GMB may affect breast health through several mechanisms, such as the modulation of inflammation, immune response, estrogen levels, and hormone metabolism. Moreover, an elevated risk of BC has been linked to dysbiosis or imbalance in the GMB [20]. Thus, understanding this axis is important for unraveling the complicated relationship between breast and gut health.

In the last few years, several studies have been performed to study the change in breast TME microbiota compared to normal persons. Urbaniak [21] analyzed breast tissue from 81 women who had cancer in Ireland and Canada. After using 16S rRNA sequencing, several taxa were found to be abundant in Canadian samples, such as *Acinetobacter* (10.0%), *Bacillus* (11.4%), *Pseudomonas* (6.5%), *Enterobacteriaceae* (8.3%), *Propionibacterium* (5.8%), *Staphylococcus* (6.5%), *Gammaproteobacteria* (5.0%), *Comamonadaceae* (5.7%), and *Prevotella* (5.0%). On the other hand, in the Irish samples, the same taxa as Canadian samples were found to be the most abundant but with different percentages: *Staphylococcus* (12.7%), *Enterobacteriaceae* (30.8%), *Pseudomonas* (5.3%), and *Propionibacterium* (10.1%).

Another study [22] was conducted in 2018 to identify different microbiome patterns in the breast tissues of Chinese cohorts of women who have cancer from those who have benign breast disease. Furthermore, profiles of the microbiome in malignant breast tissue across three distinct histological grades were compared. The results revealed that there were differences in the microbiota composition of malignant and benign tissues at the levels of phylum and family. In terms of phylum, *Proteobacteria* comprised the majority of bacteria (37.55% vs. 31.77%), *Actinobacteria* (23.2% vs. 21.9%), *Firmicutes* (22.56% vs. 26.36%), and *Bacteroidetes* (14.57% vs. 17.53%). Also, at the levels of genus and family, malignant tissues were abundant with the genus *Propionicimonas* and five families including *Rhodobacteraceae*, *Micrococcaceae*, *Methylobacteriaceae*, and *Nocardioidaceae*.

Further, to reveal microbiome differences across three distinct histological grades in malignant tissues, 56 samples of malignant breast tissue were analyzed, and other samples were stratified into grade I, grade II, and grade III. According to the Shannon index, there is no significant difference among these three grades. However, compared to grades I and II, grade III tissues showed greater alpha diversity. Also, despite the similarity in overall microbiome composition among the three histological grades, the abundance of the *Bacteroidaceae* family was reduced with increasing malignancy (grade III 6.9% vs. grade II 7.2% vs. grade I 10.4%). However, at the genus level, the result showed that *Agrococcus* abundance was elevated with increasing malignancy [22].

## 3. How Does GMB Affect Breast Tissue?

Microbiota composition has direct and indirect effects on BC. Multiple pieces of evidence from the literature were gathered from studying tumor microenvironments (TMEs) and comparing them with normal breast microenvironments. In a study by German et al. [23], which included 403 healthy women and 76 BC patients, sequencing the nine hypervariable 16S rRNA gene regions (V1V2, V2V3, V3V4, V4V5, V5V7, and V7V9) generated microbiome profiles. A total of 190 normal breast tissue samples were analyzed using transcriptome analysis. The Tyrer–Cuzick risk model was used to calculate the risk score for BC. Amplicon sequencing of the V1V2 was more suitable for the study of the normal breast microbiome. The most normally abundant families in the breast were *Acetobacterraceae*, *Lactobacillaceae* (*Firmicutes* phylum), and *Xanthomonadaceae* (both *Proteobacteria* phylum).

On the other hand, *Ralstonia* (*Proteobacteria* phylum) was histologically more common in both breast tumors and normal tissues adjacent to malignant tumors [23]. Firmicutes and Bacteroidetes, the major phyla of the colon, can influence diseases related to obesity, which are also risk factors for breast cancer. Therefore, the Firmicutes/Bacteroidetes (F/B) ratio was analyzed in patients with breast cancer. The F/B ratio was three times lower in patients with breast cancer than in healthy controls. In addition, the risk factor for breast cancer, such as fasting serum glucose, was found to be related to the F/B ratio. The F/B ratio can be used as a risk factor for breast cancer and as a clue to explain underlying mechanisms affecting the development of breast cancer [24].

Furthermore, in a study by Urbaniak et al., *E. coli*, *S. epidermidis*, and *B. cereus* strains that have been extracted from gingival plaque resulted in carcinogenic effects. They could metabolize the hormone progesterone into 5 alpha pregnane-3,20-dione (5P) [25]. 5P is more common in breast tumors than in normal breast tissue [26] and is known to stimulate cell proliferation, promoting tumor development. Moreover, Ma et al. found that the relative abundance of *Firmicutes* such as fecal bacterium with multiple types of phosphorylcholines that result from lipid upregulation was decreased, which was negatively correlated with BC, while the amounts of *Verrucomicrobla*, *Proteobacteria*, and *Actinobacteria* were increased in breast cancer patients [12]. Also, Su, Li [27] found that *Ruminococcus* was highly negatively linked to the quantity of fructose-6-phosphate within BC if its amount increased upon treating the mouse model with Paclitaxel combined with polysaccharide originating from *Ganoderma lucidum* spore (SGP), affecting the gut microbiota and tumorigenesis. In addition, An, Kim [28] investigated the effect of *Klebsiella* extracellular vesicles with tamoxifen endocrine therapy in MCF7 cells. The EVs increased therapy efficiency. Moriwaki, Begum [29] also found that the comparative profile inter-library of the SAGE-tag points out that the expression regulation could be affected by a hormone present in the reproductive tissues in humans, as proved in LNCaP with DHT versus LNCaP without 5a-dihydrotestosterone or MCF7 3h versus MCF7-estradiol 3h. Data resulting from SAGE demonstrate that the transcript from *Mycobacterium bovis Bacillus* could be expressed differentially in malignancies such as ovarian, prostate, and breast cancers.

## 4. Manifestation of Estrogens in BC

BC classification is usually based on the availability of receptors responding to estrogen (ER), progesterone (PR), and human epidermal growth factor receptor-2 (HER2). There are four subtypes according to immunohistochemistry: luminal A, luminal B, HER2-positive, and triple-negative [30]. Estrogen types are known to be a reason for the genotoxic effect by influencing a non-estrogen receptor-α-dependent mechanism [31]. The best clinical prognosis is observed in the luminal A subgroup, which has expression of estrogen receptor (ER) and activity resulting in effective hormone therapy. Downregulated ER expression and higher proliferation marks luminal B cancers. The EGFR2 (HER2)/positive subgroup lacks ER and PR complexes in about fifteen percent of total invasive breast cancers. This type is more combative than tumors of the luminal-like type. Finally, TNBCs are the most difficult to treat, thus having the worst survival rates [32]. Increased exposure to estrogens affects hormonal-dependent cancers, which compose more than 70% of all BCs, as the onset, progression, and treatment of cancer are affected by hormones. Estrobolome influences hormonal balance and is linked to an elevated risk of developing breast cancer [33].

Microorganisms and the host live in symbiosis and play an important role in preserving homeostasis. The microbiota participates in several metabolic pathways, including fermentation, absorption of not completely digested carbohydrates, assistance in energy harvesting, storage, and the regulation and activation of the immune system [34]. Immune infiltration in the TME modulates estrogen metabolism [32].

**Figure 1:**
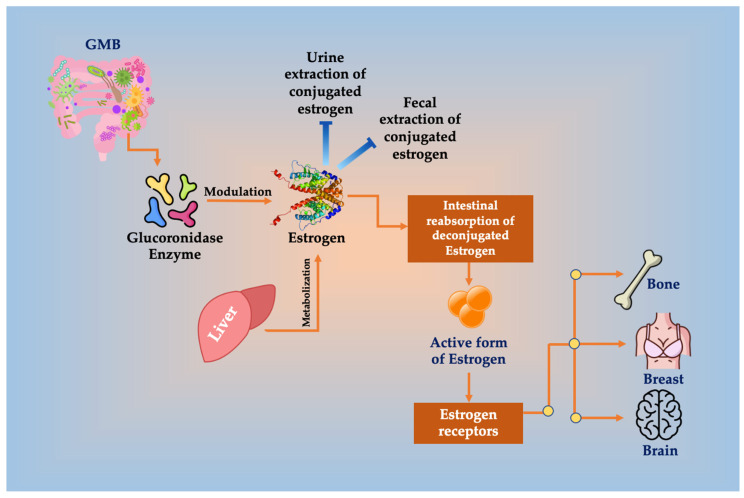
Glucuronidase enzyme of GMB deconjugates estrogen, resulting in the inhibition or decreased conjugated estrogen extraction in urine and feces, enhancing its reabsorption in the intestines and increasing the amount of the active form of estrogen that binds to ERs in bone, brain, and breast tissues, increasing the risk for BC.

The liver metabolizes estrogens. Redox reactions in the gut and inducing synthesis of estrogen-inducible growth factors produce similar metabolites to estrogen, which might have carcinogenic potential [35]. The metabolites are conjugated and excreted into the bile to GIT lumen, where de-conjugation by bacterial β-glucuronidase occurs. Bacterial β-glucuronidase enzymes are divided into two dominant subgroups, the *Clostridium leptum* cluster and the *Clostridium coccoides* cluster of the *Firmicutes phylum*. *Escherichia*/*Shigella* of the *Proteobacteria* phylum likewise has β-glucuronidase enzymes [36]. Xenobiotics and/or xenoestrogens de-conjugation are completed, leading to their reuptake in the entero-hepatic pathway [35]. In the intestines, microbes can metabolize polyphenols and phytochemical compounds derived from plants to synthesize estrogen-like compounds [37] (Figure 1). Then, free estrogens are re-absorbed through enterohepatic circulation, reaching breast tissue [15]. All the proliferative pathways are initiated by the active metabolite 27-hydroxycholesterols, which has a chemical structure similar to estrogen and can bind to hormone receptors. It originates from large amounts of cholesterol that are transformed by the enzyme CYP27A1 [38].

### 4.1. Estrogen Action: The Nuclear and Non-Nuclear Mechanisms

Cellular mechanisms affected by estrogen can include nongenomic cellular signaling, direct DNA binding, ligand–receptor binding, activation, receptor-mediated nonligand hormone activities, gene regulation, and non-DNA binding [39]. Estrogen aids in the development of BC by generating oxidative DNA damage resulting from genotoxic metabolites and ER signaling [40]. The GeneMANIA online tool found a direct association between 19 DNA damage repair (DDR) genes with both BRCA1 and ER-α, which regulate mammalian DNA damage repair [41]. Estrogen and the ER complex directly bind to the regulatory DNA elements (EREs), eliciting more factors affecting transcriptional regulation. ER can also regulate transcription by binding indirectly to Sp1 or activator protein-1 (AP1) binding sites. ER is phosphorylated by growth factors (IGF and EGF) as they bind to membrane growth factor receptors (GFRs) that regulate gene expression, recruiting intracellular signaling pathways, although ligands are absent. Membrane ERα or GPR30 also binds to and is activated by estrogen, rapidly inducing the intracellular signaling pathway [39].

### 4.2. Menopausal Hormonal Imbalance

Adiposity is the gain of fat after menopause due to hormonal changes. It increases the risk for BC after menopause. How BC is treated and responds to hormone-based therapy in premenopausal differs from menopausal and postmenopausal women. Menopause affects estrogen levels and breast microbiota composition. It is well-known that adjuvant chemotherapy is of greater benefit in premenopausal women compared to postmenopausal women [42,43]. The reason underlying this is that the composition and functions of GMB are not similar in postmenopausal BC patients and healthy controls. Zhu and his colleagues indicated that 45 species varied significantly between postmenopausal controls and postmenopausal patients; of them, 38 species were enriched in postmenopausal patients, including *Klebsiella* sp., *Enterococcus gallinarum*, *Escherichia coli*, *Prevotella amnii*, *Actinomyces* sp. HPA0247, *Shewanella putrefaciens*, and *Erwinia amylovora*, and 7 species were less abundant in postmenopausal patients, including *Eubacterium eligens* and *Lactobacillus vaginalis* [44].

### 4.3. Targeting Estrogen-ER Signaling

A study by Polkinghorn et al. has identified 32 genes responsible for DNA repair that respond to activated AR in xenografts of the androgen-sensitive human prostate adenocarcinoma cell line (LNCaP), proving the occurrence of cell communication between the DNA repair regulation and the AR pathway [45]. The dynamics of DNA strand breaks generated by induced estrogen include a two-phase pattern and DNA strand breaks mediated by topoisomerase. It has been proved that DNA oxidation in the late phase results from ERa-dependent transcription, which was not documented in tamoxifen treatment alone [46]. Regarding ER^+^ BC, Xie, Zahid [40] examined 17β-estradiol (E2) in the presence or absence of selected Keap1-Nrf2 protein–protein interaction (PPI) inhibitors. Keap1-Nrf2 PPI inhibitors downregulated the mRNA and protein levels of estrogen induced by E2 exposure responsive genes on ER^+^ BC cells, MCF7. The outcomes implied that the Keap1-Nrf2 PPI inhibitors have possible antioxidant activity by activating Nrf2 pathways and inhibiting E2-induced gene and protein expression, serving as potential agents for reducing the development of BC [40]. Several studies have been conducted on targeting the BC microenvironment using natural products and active compounds that can affect the GMB, resulting in enhanced treatment of BC, such as Berberine and Genistein. Berberine involving GMB and endogenous metabolites under a hypoxic microenvironment inhibited the proliferation of the ER^+^ BC cells MCF7. Transwell invasion and wound healing studies indicated that berberine inhibited the invasion and migration of BC cells by affecting the HIF-1α. In contrast, the inhibitory effect of berberine was not affected by the hypoxic microenvironment [47,48]. Moreover, Genistein is a phytoestrogen that inhibits the proliferation and differentiation of MCF7, the BC cells that express ER and PR. The possible mechanism behind this process is the downregulation of the Sonic Hedgehog-Gli1 pathway, which is responsible for developing chemoresistance and radioresistance. Genistein is favorable in the case of treating BC in postmenopausal women, as it binds with a larger affinity to Erβ, resulting in an antiproliferative effect [49].

## 5. The Microbiota–Breast Axis: Bidirectional Action

**Figure 2:**
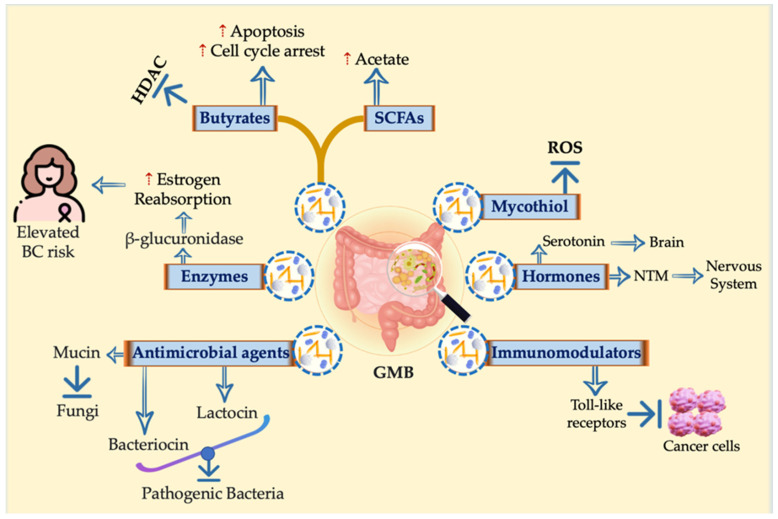
Holistic view for different molecules with various biological activities generated by the GMB. Different molecules with various biological activities are generated by the GMB, including SCFAs such as propionate, butyrate, and acetate; secondary bile acids; enzymes such as glucuronidase that affect estrogen metabolism and may cause cancers, especially breast cancer; antimicrobial effects such as bacteriocin; immune modulators that prevent tumorigenesis; and some hormones that act as neurotransmitters.

The GMB and the host immune system work closely together to protect against and combat infections, playing a crucial role in maintaining human health overall. Metabolites generated via the GMB population have a bidirectional effect on host hemostasis [50] (Table 1). The GMB confers numerous positive effects and is intricately connected to an individual’s overall health condition. However, our GMB can also contribute to the development of diseases, including an increased susceptibility to cancer [51]. The GMB generates different molecules with various biological activities, including SCFAs (such as propionate, butyrate, and acetate), secondary bile acids, enzymes (such as glucuronidase that affect estrogen metabolism and may cause cancers, especially breast cancer), antimicrobial substances (such as bacteriocin and lactocin), immune modulators that prevent tumorigenesis, and some hormones that act as neurotransmitters (Figure 2).

SCFAs are some of the substantial metabolites generated by GMB. Among the metabolites found in the human gut, this group stands out as the most abundant, with butyrate being the predominant *Firmicutes*-produced SCFA. A substantial body of evidence supports the role of SCFAs in preserving intestinal health and alleviating various types of malignant tumors. In premenopausal BC patients, SCFAs play a critical role in the underlying pathological mechanisms, with butyric acid showing the most substantial reduction among the BC group [50].

Butyrate may have a specific role in mitigating the advancement of BC cells. The prevailing belief is that the primary mechanisms by which SCFAs exert their effects are as follows: the first mechanism involves direct absorption via intestinal epithelial cells into the bloodstream. Once in circulation, they bind to the G protein–binding receptor (GPR) on immune cells, thus influencing immune regulation and the modulation of inflammatory factors. GPCRs that are SCFA receptors imply that they are involved in several cellular pathways [52]. Macrophages, adipocytes, and colonocytes all have GPR109A as a surface receptor. An additional risk factor for CRC advancement is a decrease in GPR109A expression. T reg cell development and the production of IL-10 and IL-18, two cytokines that promote and inhibit inflammation, are supposedly aided by GPR109A. As shown in Niacr1-/-mice, these reactions have been linked to carcinogenic effects. SCFAs may mess with cell cycle control and cell death. In MCF-7 cells, activation of GPR41/43 upregulates intracellular Ca2+ levels and stimulates mitogen-activated protein kinase (MAPK) p38 [53]. There is strong evidence connecting these findings to cellular stress responses and cancer development [54].

The second involves SCFA conversion into acetyl-CoA within the cell. This process leads to an increase in ATP/ADP levels and activates the mTOR signaling pathway, ultimately regulating the activity of T cells. The third mechanism attributed to SCFAs involves their ability to inhibit histone deacetylase (HDAC) activity. This inhibition leads to increased acetylation of histones and other protein elements [55]. SCFAs like butyrate could diminish the BC cell invasion capabilities by employing diverse signaling mechanisms like proliferation pathway modulation. By inducing a transition of cells from an invasive mesenchymal phenotype to a quiescent epithelial phenotype, SCFAs hold the ability to hinder tumor metastasis.

**Table 1:** The effect of gut microbiota metabolites on different metabolic pathways that affect breast cancer directly or indirectly.

Metabolites	Metabolic Pathway	Effect	Reference
Secondary bile acids (Lithocholic acid)	Bile acid metabolism	- Oxidative stress ↑ - NRF2 expression ↓ - Apoptosis in BC ↑ - BC cell proliferation ↓ - Anti-tumor effect ↑ - Good prognosis ↑	[56]
β-glucuronidase enzyme	Estrogen metabolism	- The active form of estrogen ↑ - Level of estrogen in the bloodstream ↑ - BC progression ↑	[57]
SCFAs (butyrate)	Immune pathway	- Treg cells proliferation ↑ - Prostaglandin E_2_ ↑ - Production of cytokine CCL_22_ ↑ - Altering immune pathways ↑	[58]
	Epigenetic level	- Activate silenced genes in cancer cells, such as p21 and BAK ↑ - HDAC inhibitor ↑	[55]

Within the TME, SCFAs can improve the competitiveness of CD8^+^ T cells against tumor cells for glucose resources. This, in turn, promotes the CD8^+^ T cells’ survival and activation. Metabolic and epigenetic reprogramming induced by SCFAs enhances the anti-tumor activity of CD8^+^ T cells and chimeric antigen receptor (CAR) T cells [59,60]. Thus, SCFAs could modulate the T cells’ metabolism based on the host’s conditions.

The study of the impact of intestinal flora on both health and disease has emerged as a prominent research area. The diversity, composition, and metabolic processes of the gut microbiota play a crucial role in generating protein fermentation metabolites. Toxicity is associated with various protein fermentation products, like amines, polyamines, phenols, indoles, carnitine, and hydrogen sulfide (H_2_S) [56].

Several species of GMB can produce H_2_S via cysteine degradation, including *Bacteroides*, *Escherichia*, *Enterobacter*, *Clostridium*, *Collinsella*, *Fusobacterium*, *Klebsiella*, *Prevotella*, *Proteus*, and *Streptococcus*. Hydrogen sulfide (H_2_S) has recently been recognized as belonging to the well-characterized gaseous biological mediators, termed gasotransmitters, where it plays a pivotal role in regulating different oncogenic signaling pathways (both canonical and non-canonical), including PI3K/AKT/mTOR [61], JAK/STAT [62,63], Ras/Raf/MEK/ERK [61], and nitric oxide (NO) [63] signaling cascades. Moreover, the non-coding RNA (ncRNA) machinery in BC cells undergoes regulatory effects mediated by H_2_S. The inhibition of the enzymes responsible for H_2_S synthesis suppresses the BC oncogenicity, as evidenced by its impact on cell proliferation, invasion, migration, and survival. It enhances their responsiveness to both adaptive and innate cellular immunity [64].

Secondary bile acids are all microbial metabolites. The intestinal microflora facilitates the conversion of primary bile acids into secondary bile acids through the processes of dehydrogenation and de-conjugation. Bile acids have been described to reduce BC proliferation, contrary to gastrointestinal cancers [65,66]. Studies have documented the anti-tumor effects of bile acids on BC, demonstrated in cell lines and patient tissues. Lithocholic acid (LCA) triggers oxidative stress and apoptosis throughout multiple pathways, such as *TGR5* activation and *NRF2* downregulation [67]. Apart from its role in regulating oxidative phosphorylation, LCA also elicits anti-tumor immune responses while simultaneously suppressing proliferation and metastasis [66].

A low bile acid metabolism breast TME harboring microorganisms is linked to aggressive cancer characteristics, such as enhanced cell proliferation and unfavorable survival outcomes [56]. In contrast, elevated bile acid metabolism in cancer cells leads to the induction of apoptosis, ultimately improving patient survival and yielding a positive prognosis.

## 6. Breast Cancer Hypoxia: The HIF Signaling Pathway

### 6.1. Hypoxia Effects on Genes

The dividing cells need glucose, amino acids, oxygen, and reducing equivalents (for example, NADPH) for energy and biomass synthesis [68]. The oxygenation in healthy tissues, referred to as tissue normoxia or hypoxia, varies greatly between the organs as a result of a diverse blood artery network and metabolic activity [69]. Hypoxia, which is characterized by low oxygen levels, is an essential state for many physiological processes, including the healing of wounds [70]. When a tissue’s oxygen concentration falls below what is needed for cellular life, it is referred to as hypoxia [71]. The majority of solid tumors possess hypoxia, which is a feature of the TME that is linked to the development and spreading of cancer [72]. A partial O_2_ pressure (PO_2_) of less than 10 mmHg is referred to as a hypoxic TME [73]. Comparable to the PO_2_ of approximately 65 mm Hg in normal breast tissue, the median PO_2_ in BC is approximately 10 mm Hg [74]. Tumor acidosis is the outcome of cancer cells’ abnormal metabolism. In solid tumors like BC, this is frequently observed. The low pH levels influence cancer cells and have also been demonstrated to inhibit immune cells in tumor acidosis. By generating an aggressive phenotype and reducing immune cell activity, the low pH of the surrounding tissue is known to facilitate metastasis and proliferation [75]. According to earlier research, an acidic environment encourages tumor cell metastasis [76], and a hypoxic TME can increase tumor resistance [77]. Comprehensive study and efficient management of the TME will yield efficient methods for treating tumors, as it is a crucial factor in the growth of tumors [78,79].

Meanwhile, tumors that have hypoxic areas develop because of excessive O_2_ consumption brought on by either an insufficient O_2_ supply or an increase in the proliferation of tumor cells. The disorderly, inadequate tumor vascular network, which is characterized by leaky arteries unable to make up for the O_2_ shortage, is the cause of this inefficient O_2_ supply [73]. Another factor that promotes hypoxia in solid tumors is the tumor’s distance (of around 100 μm) from functioning arteries [80].

Hypoxia promotes a complicated cell signaling pathway in cancer cells, which includes the MAPK, PI3K, NFĸB, and HIF pathways, which connect generating negative and positive feedback loops and boosting or lessening hypoxic effects [81]. In tumor cells, hypoxia-inducible factor-1 (HIF-1) plays a very important role in controlling the hypoxic reaction. HIF is composed of two subunits: HIF-1α (120 kD) and HIF-1β (91-94 kD). Oxygen concentrations regulate the activity of HIF-1, which is determined by the α subunit [82]. In a hypoxic state, tumor cells can become more resistant to drugs and capable of metastasizing when their growth outpaces the oxygen supply from the capillaries inside the tumor [83]. Thus, it has been suggested that the major challenge to achieving a complete cure for malignancies is effectively diagnosing and treating hypoxia in tumors [84]. Genes that cause cancer to invade and spread are expressed variably in tumor cells when there is less oxygen present [85].

**Figure 3:**
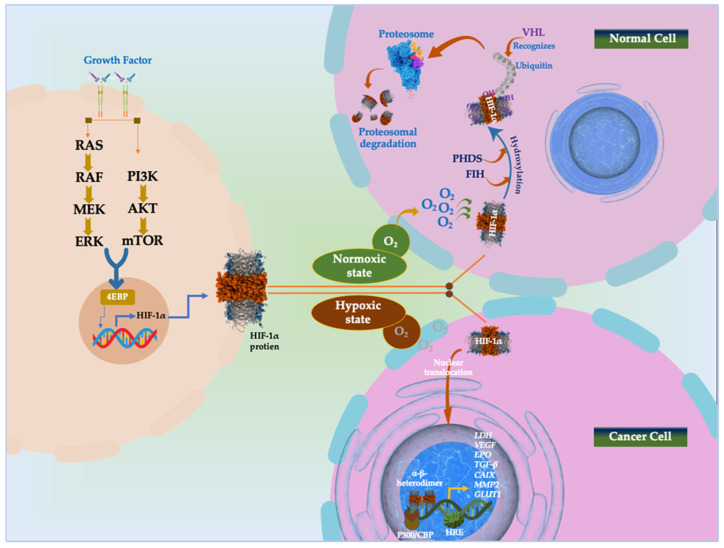
HIF signaling pathway in normoxic and hypoxic state. Phosphatidyl inositol-4,5-bisphosphate-3-kinase (PI3K)-protein kinase B (PKB)/AKT pathway; co-activator CBP/p300; mammalian target of rapamycin (mTOR); MAPK/extracellular signal-regulated kinase (MEK); extracellular signal-regulated kinase (Erk); eukaryotic translation initiation factor 4E (eIF-4E) binding protein (4E-BP1); asparagine 803 (Asn803); factor-inhibiting hypoxia-inducible factor (FIH); Vascular Endothelial Growth Factor (VEGF); erythropoietin (EPO); Lactate Dehydrogenase (LDH); Transforming Growth Factor β (TGF-β); Carbonic Anhydrase 9 (CAIX); Matrix Metallopeptidase 2 (MMP2); Glucose transporter 1 (GLUT1); tumor-suppressing protein von Hippel–Lindau (pVHL); hypoxia response elements (HRE).

Helix–loop–helix proteins, also known as HIFs, are heterodimeric complexes consisting of an O_2_ dependent α-subunit (HIF-1α, 2α, and 3α) and a constitutively generated β-subunit (HIF-1β) [86]. Under normoxic environments, the von Hippel–Lindau (pVHL) are bound by the HIF-1α conserved proline residues, which then undergo hydroxylation by prolyl-hydroxylases (PHDs) and catalyze its ubiquitination-dependent proteasomal destruction [73]. Once oxygen is depleted, however, HIF-1α could assemble and transfer to the nucleus, where it forms a heterodimer with HIF-1β due to the suppression of PHDs (Figure 3). After binding to the hypoxia-responsive element (HRE) and the transcriptional coactivator p300/CBP, the heterodimer HIF-1α/HIF-1β stimulates the transcription of the HIF target gene [87]. The activation of several particular genes that control several tumor biological processes, including epithelial and mesenchymal tissue transition (EMT) and angiogenesis, the immune system, metabolic reprogramming, invasion and metastasis, and the survival and multiplication of tumor cells, is made possible by hypoxia-dependent HIF-1α and HIF-2α [87].

Factor inhibiting HIF-1 (FIH) is an additional factor that hydroxylates HIFs at Asn803 to reduce HIF-α transcriptional activity. When hypoxia happens, PHD and FIH activities decrease, stabilizing the HIF-α protein. After that, HIF-α moves to the nucleus and joins forces with HIF-β to form a heterodimer [88,89]. After attaching to the promoter of genes containing HREs, the HIF-α/β heterodimer activates genes involved in several normal and pathological pathways [88,90].

### 6.2. Targeting HIF-1α

Berberine, a benzylisoquinoline alkaloid, has been found to effectively suppress BC growth and metastasis in a hypoxic TME. The study found that berberine altered GMB profusion and diversity in mice with BC, leading to a higher survival rate and representing a potential basis for berberine treatment in BC patients. Berberine can inhibit BC growth and metastasis by affecting the expression of E-cadherin, β-catenin, and N-cadherin in highly metastatic BC cells and potentially by affecting tumor cells under hypoxic conditions [47].

Cytotoxic effects of both *Lactobacillus crispatus* and *Lactobacillus rhamnosus* (LCS and LRS) on MDA-MB-231 cells indicate that LRS would be preferable for therapy that is pathway-directed due to its downregulation of HIF-pathway-mediated oncogenes. LCS’s inhibitory effect on HIF-1α and HSP90 expression suggests its potential for use in cancer treatment strategies [91].

Anaerobic bacteria, including *Bifidobacterium*, *Clostridium perfringens*, *Salmonella*, and *E. coli*, can selectively colonize deep hypoxic tumors, potentially acting as carriers for chemotherapeutic drugs [92]. The study created the (Bif@DOX-NPs) platform, a biocompatible hybrid of bacteria and nanoparticles that delivers adriamycin-loaded bovine serum albumin nanoparticles into BC using anaerobic *Bifidobacterium infantis*. The biohybrids actively colonize hypoxic tumors, increasing drug accumulation and prolonging median survival in mice. Biohybrids made of anaerobic bacteria show promise for the targeted therapy of tumors [93].

Inosine, a metabolite produced by *Bifidobacterium pseudolongum*, is primarily concentrated in the small intestine’s duodenum and diminishes down the gastrointestinal tract. Its impact on immune checkpoint blockade therapy efficacy and the ability of some inosine-producing bacteria to boost immunity suggests its potential as a natural anticancer agent [94]. Another study indicates that inosine is the primary cytoprotective component in BC hypoxia, challenging the earlier theory of adenosine as the key compound [95]. To combat drug resistance and comprehend its mechanisms, more research is required [96].

Angiogenesis in tumors leads to irregular blood vessels, insufficient oxygen supply, and hypoxia or anoxia regions in malignant tumors [97]. Bacteria like *Bifidobacterium*, *Clostridium*, *Salmonella*, and *Escherichia* colonize tumors, while obligatory anaerobes survive in anoxic areas [98]. Synthetic biology has recently acknowledged the potential of bacterial therapy for tumor treatment, as tumor-seeking bacteria can synthesize various therapeutic agents [99]. It has been discovered that the pore-forming protein Hemolysin E (HlyE), which is present in *E. coli*, is extremely cytotoxic to cancer cells and can permeate deeply into tumor tissue. Its high cytotoxic activity has led to its application in cancer therapy, with studies showing increased necrosis of 4T1 BC cells and marginal affection of 4T1 breast murine tumors [100]. The study uses a xenograft tumor model to study tumor immunotherapy effectiveness. Tumor-immunosuppression-related genes can be knocked down using the CRISPR/Cas9 system. An autonomous, multipurpose delivery vector, *Lactobacillus rhamnosus GG*, is used to distribute the CRISPR/Cas9 nanosystem to the tumor, promoting immune responses and preventing tumor re-challenge in vivo [101].

## 7. Immune Modulation: The Role of GMB

Our GMB has special pathogen-associated molecular patterns (PAMPs) conserved within a class of microbes. These molecular motifs are recognized by Toll-like receptors (TLRs) which are a family of *pattern recognition receptors* found on the surface of various immune cells and considered an elementary component in the immune system. This recognition leads to the initiation of an immune response. There may be a direct correlation between TLRs and breast cancer. According to new research, each cell line has different Toll-like receptor expression, and the expression level may differ in the same line. TLR2 in MCF7 cell lines has a lower expression level than MDA-MB-231 [102]. TLRs, whether signaling through (MyD)88-dependent pathway or (MyD)88-independent pathway, activate interferon regulatory factor (IRF), activating protein-1, which leads to the development of pro-inflammatory cytokines (IL-1, IL-6, IL12, TNF-α, and type-1 IFNs [103].

The GMB not only affects the immunity in BC by its molecular pattern but also produces some metabolites that affect immunity; these metabolites include propionic acid, acetic, and butyric acid, which are the major SCFAs [104]. There is an accumulation of immune cells including transforming-growth-factor-β-producing cells such as leukocytes, T cells, and interleukin-10, which result from binding GPR43 and GPR41 to SCFAs; GPR43 and GPR41 are examples of G-protein-coupled receptors found in the immune cell membrane and have an affinity to SCFAs [105]. Managing gene expression is the key point in the activation of macrophages, which is regulated by any modification or transcription factor. Histone acetylation alters the chromatin to be relaxed and transcriptionally active, which exposes the cells to any modification or regulation [106].

Beneficial *E. coli* found in our guts have five strains that produce SCFAs, and these strains are non-pathogenic. One of them is *E. coli* KUB-36, which has a role in reducing inflammation according to its metabolites [107]. *E. coli* has lipopolysaccharide, an endotoxin that is responsible for the toxicity of *E. coli* consisting of lipid A and a core polysaccharide. This LPS was responsible for inducing the expression of pro-inflammatory cytokines like IL-6, IL-1β, TNF-α, and IL-8 [107]. Activation of the NLRP3 inflammasome by LPS+ ATP is similar to widespread metabolic abnormalities in macrophages. To restore metabolic function, PDHK inhibition increased glucose absorption and flow via glycolysis and the TCA cycle via PDC and PC anaplerosis portals, allowing cells to produce more energy and survive [108].

Trimethylamine N-oxide, also another microbial metabolite, can induce pyroptosis in triple-negative BC cells [109]. Clostridiales produce TMAO because they create the precursor of TMAO, which is trimethylamine (TMA) [110]. To confirm the role of trimethylamine N-oxide in a BC mouse model, mice were injected with TMAO, showing that it can suppress tumor growth by enhancing the function of immune cells found in the TME like M1 macrophages and CD8^+^ T cells such as tumor necrosis factor-α, with interferon-γ found in high levels. Other studies suggest that the CD8^+^ T may be changed or improved because of our commensal microbiota [55]. The other proof of TMAO involvement is pyroptosis, and it was found that TMAO can affect the tumor cells directly. A cell death/cytotoxicity assay was used to assess the effect of TMAO LDH along with a PI assay, which confirmed that cells undergo pyroptosis [111]. Two proteins involved in pyroptosis were detected at high levels by Western blotting: GSDME and cleaved caspase 3 [109].

## 8. Chemoresistance and Microbial Metabolites

Many microbial metabolites ameliorate the aggressiveness of BC, such as cadaverine, which is used as a treatment for BC. Cadaverine helps promote metastasis and cell movement and inhibits cancer cell growth in the 4T1 cell line through elevated expression of some receptors [112]. However, not all microbiota metabolites help treat BC; they also may affect drug metabolism and confer resistance to certain types of them. There is a bidirectional relationship between the microbial metabolites and drug metabolism. Alexander et al. [113] illustrated the effect of GMB on drugs and suggested that multiple pathways influence drug metabolism by modulating the activity of drug-metabolizing enzymes in the liver and other tissues or influencing TME by regulating immune cell balance and translocation.

The importance of knowing the species that affect the metabolism of the drug helps in reversing its effect; we can add antibiotics for the species that interfere with the efficacy of the chemotherapy drugs. *Gammaproteobacteria* produce cytidine deaminase (CDDL), an enzyme that degrades Gemcitabine, one of the drugs used in BC chemotherapy, into its inactive form (difluoro-deoxy-uridine) [114]. The administration of ciprofloxacin antibiotic, which targets *Gammaproteobacteria* and other gut bacteria along with gemcitabine, may increase the efficacy of the drug and prevent the effect of cytidine deaminase [114].

Irinotecan is a drug used in the treatment of BC and many different types of cancer [115]. Some bacterial species, such as *Clostridium* and *Bacteroides*, produce β-glucuronidases, which induce adverse effects of the drugs. Irinotecan transforms to its active form (SN-38) once taken, and it then transforms to SN-38G, which is a glucuronidated form, or the inactive form SN-38, and this inactivation occurs by UDP-glucuronosyltransferase. The role of the bacteria and β-glucuronidases produced by it is to recognize SN-38G as a source of carbon and react with this glucuronidated form. This conversion and reactivation induce adverse effects of the drug [116].

Capecitabine has been reported to be used in the treatment of BC and also in first-line chemotherapy with a combination of other drugs [117]. Capecitabine works as an antimetabolite, inhibiting DNA replication in cancer cells by reducing thymidylate synthase, which is a crucial enzyme in the production of thymidylate, a necessary precursor for the synthesis of DNA [118]. The relationship between Capecitabine deglycosylation and uridine phosphorylase, an enzyme produced by some GMB like *E. coli* and *Salmonella typhimurium* [119], has been proven, which induces resistance to Capecitabine using screening and metagenomic analysis methods [120].

GMB activates or inactivates chemotherapeutic medicines. Drug effectiveness depends on the gut microbiome. However, Chiba et al. found that neoadjuvant chemotherapy may modify the breast microbiota. In breast tumor tissue, the *Pseudomonas* spp. abundance increased while bacterial diversity decreased. Prevotella in tumor tissue was similarly reduced in non-treated patients [18].

Viaud (2015) found that the gut microbiota affects the effectiveness or toxicity of cyclophosphamide, platinum salts, and irinotecan. The gut mucosa may be damaged by cyclophosphamides, allowing gut microbes to enter the circulation. However, Lactobacillus plantarum HY7712 in the gut microbiota protects mice against cyclophosphamide-induced immunosuppression [121]. Streptomyces WAC04685, a gut bacterium, deglycosylates doxorubicin to inactivate it [58,122]. Immunomodulator medications, which have been shown to be effective in cancer therapy, may alter patients’ microbiomes, and their characterization may shed light on disease responses to treatment. We must also study how various antibiotic regimes affect the breast/gut microbiome and BC development in mice models and BC patients.

Fecal microbiota transplantation (FMT) may improve immunotherapy and lessen chemotherapy side effects by changing gut flora. To determine the best cancer treatment, FMT must be examined for safety, duration, dosage, formulation, distribution, and combinations [123].

FMT modifies the gut microbiota, the host’s immune system, tumor resistance, and adverse effects to increase monotherapy effectiveness. FMT applications may transfer viral infections that may induce diarrhea, cramping, bloating, gas, constipation, and low-grade fever in immunosuppressed individuals [124]. However, gut bacteria may impact cancer therapies, notably immunotherapy [125]. FMT may enhance anticancer therapy and lessen side effects. However, further research is required to establish its safety alone or in conjunction with cancer medicines by addressing potential side effects and possible solutions.

## 9. Lifestyle and GMB

### 9.1. Diet

Dietary fibers are complex carbohydrates found in plants (such as fruits, vegetables, legumes, nuts, seeds, and grains) that can be selectively metabolized only by the GMB through anaerobic fermentation to produce SCFAs, mainly acetate, propionate, and butyrate, which are utilized as a source of energy and as well as for the synthesis of lipids. Therefore, dietary fibers can impact the GMB composition, diversity, and richness [46,126]. A daily supplement of fiber ingredients, e.g., psyllium husk and wheat bran, is added to the diet to increase the production of the butyrate-producing bacteria *Anaerostipes* [127]. In addition, levels of SCFAs (mainly butyrate) in curdlan-supplemented high-fat-diet-fed mice significantly increased, with a significantly increased level of the *Bacteroidals* S24-7 family, which is known to have propionate- and butyrate-producing properties [126]. Butyrate has presented with potential anticancer activity through different mechanisms of action against BC [128]. Supplementation of the green Mediterranean diet (Green-MED) enriched with polyphenols derived from Mankai and green tea increases the abundance of *Prevotella* and decreases *Bifidobacterium* in the GMB. Green tea contains catechins, which are flavonoid compounds, and shows strong chemopreventive and chemotherapeutic effects against BC when combined with tamoxifen or paclitaxel [129,130].

The long-term intake of different protein diets at a normal dose of 20% for 8 months alters GMB diversity in mice from 4 to 8 months by increasing the levels of the genus *Bacteroidales* S24–7, representing 31.43% of the gut microbial population. Conversely, *Rikenellaceae* RC9 gut, *Akkermansia*, *Clostridiales vadinBB60*, *Anaero truncus*, *Clostridium sensustricto* 1, and *Alistipes* were significantly reduced. Meanwhile, a meat protein diet also led to dynamic changes in the levels of total SCFAs by decreasing the level of butyrate, acetate, and isobutyrate at 4 months while increasing propionate and isovalerate levels [131].

In a human study, the consumption of pork meat protein diets reportedly causes the GMB composition in specific gut bacteria including *Lachnospiraceae* NK4A136, *Odoribacter*, *Defluviitaleaceae* UCG-011, *Ruminiclostridium* 9, *Blautia*, *Lachnoclostridium*, and *Ruminococcaceae* UCG-010, which regulated the absorption and secretion of proteins in the gut [132]. In contrast, the uptake of a low-carbohydrate, high-protein (LC/HP) diet from healthy dietary sources improved the GMB composition by decreasing the levels of bacteria related to cardiometabolic disorders such as *Tyzzerella*, *Phascolarctobacterium*, *Romboutsia*, *Clostridium sensu stricto* 1, *Hungatella*, *Ruminococcus gauvreauii*, and *Bacillales*, and also increased the abundance of *Bacteroides thetaiotaomicron*, *Coprococcus* 3, *Fusicatenibacter* and *Tannerellaceae* in patients with chronic spinal cord injury [133].

Excessive consumption of a fast food (FF) diet, including burgers, fries, and soft drinks, is associated with increased *Collinsella*, *Parabacyteriodes*, *Escherichia*/*Shigella*, and *Bilophila*. *Butyricicoccus* and *Lachnospiraceae-UCG-004* were increased after consumption of the Mediterranean (Med) diet, which is rich in vegetables, whole grains, olive oil, nuts, and fish. As a result of variation in human GMB composition, several indole derivatives decreased after FF and increased after following a Med diet, whereas tryptophan, indole-6-carboxaldehyde, and 4-(1-piperazinyl)-1H-indole increased after FF diet and decreased after Med diet [134].

High consumption of 750 g/week of cod or salmon fillet affects the microbiota with lower counts for bacteria in the *Bacteroidetes* phyla and the *Clostridiales* order of the *Firmicutes* phyla. Higher counts for bacteria in the *Selenomonadales* order in the *Firmicutes* phylum were observed due to trimethylamine N-oxide (TMAO), which is found naturally in fish and certain types of seafood. The TMAO content reduces bile acid production from cholesterol in the liver [135].

### 9.2. Vitamins

Vitamin D intake in humans was associated with decreased levels of circulatory lipopolysaccharides, a component of the Gram-negative bacterial cell wall; reduced abundance of *Coprococcus* and *Bifidobacterium*; and increased levels of *Prevotella* [136]. Acute vitamin A deficiency diet leads to a bloom of *Bacteroides* in the deficient-diet-fed mouse. Also vitamin A deficiency was associated with a significant increase in tauro-β-muricholic acid sulfate at the end of the deficiency phase at experimental day 35 [137].

Thirty days of treatment with probiotics (*Bifidobacterium longum BB536* and *Lactobacillus rhamnosus HN001*) and vitamin B6 led to the enrichment of several genera involved in lactose digestion including *Bifidobacterium*. This modulation could also be the result of the intake of vitamin B6. Moreover, the relative profusion of acetic acid, 2-methyl-propanoic acid, nonenal, and indolizine 3-methyl increased, while phenol decreased [138].

### 9.3. Alcohol

Alcohol exerts numerous adverse effects on the gastrointestinal tract and its associated glands. In recent years, there has been an increasing recognition of structural alterations of the GMB. A study reported that patients with alcohol overconsumption suffer from small intestinal malabsorption and impaired colonic microbial metabolism [139]. These impaired colonic microbial metabolisms are associated with intestinal bacterial overgrowth and hyperpermeability, leading to the abundance of the phylum *Proteobacteria*, a Gram-negative-producing endotoxin, while lowering the count of butyrate-producing bacteria from the genera *Faecalibacterium*, *Sutterella*, *Holdemania*, and *Clostridium*, with a significant decrease in the production of SCFAs, particularly butyric acid [140]. Furthermore, rats that consumed 20% ethanol on alternate days for 13 weeks showed a decreased abundance of *Lactobacilli* and increased *Bacteroidetes* and Archaea *Methanosphaera* with overproduction of acetate [141]. Also, young binge drinkers were affected by several microbiome alterations, including increases in *Bacteroides* spp. and *Veillonella* and reductions in *Alistipes* spp., with reductions in butyrate, valine, and cysteine and increased acetate synthesis [142]. After consumption, ethanol primarily undergoes oxidative metabolism in the liver to produce acetaldehyde that is subsequently converted to acetate by aldehyde dehydrogenases (ALDHs) and then released from the liver [143]. In addition, alcohol is also oxidized in non-liver (extrahepatic) tissues. Acetaldehyde (AcH) is the first metabolite that is produced from the ethanol oxidation process by gut obligate anaerobic microbiota such as *Bacteroides*, *Bifidobacterium*, *Collinsella*, and *Ruminococcus* under aerobic conditions [144]. AcH is a mutagen that can form adducts with protein and DNA, causing gene mutation. Thus, acetaldehyde is classified as a Group I carcinogen [145]. BC is most consistently associated with alcohol consumption, according to the type of alcoholic beverage consumed [146,147]. According to meta-analyses, for every 10 g of alcohol consumed per day, the risk of BC increases by 2–12% in pre-and postmenopausal women [148]. This positive correlation could be associated with the ability of ethanol to affect growth factor and nutrient homeostasis as, for instance, vitamin D deficiency, an increase in oxidative stress that leads to the accumulation of acetaldehyde and production of ROS [145,149]. Moreover, the study showed 18% higher mean salivary 17-β-estradiol levels throughout the menstrual cycle among women who consumed more than 10 g of alcohol per day compared to women who drank less than 10 g of alcohol per day [150].

### 9.4. Smoking

Smoking is a major risk factor for a variety of diseases; it entails the inhalation of a mixture of complex chemicals including nicotine, aldehydes, polycyclic aromatic hydrocarbons (PAHs), nitrosamines, heavy metals, and other compounds that are inhaled into the lungs as aerosol particles or free in a gaseous state including carbon dioxide (CO_2_), carbon monoxide (CO), and hydrogen sulfide (H_2_S), which affect O_2_ transport, decrease the pH of blood, and induce inflammation diseases [151]. A study showed that gut composition in current smokers had an increased proportion of the phylum *Bacteroidetes* with decreased *Firmicutes* and *Proteobacteria* compared with never-smokers. In contrast, there were no differences between former and never-smokers [152]. An in vitro study found that exposure to cigarette smoke for 2 h/day for 28 weeks significantly increased GMB dysbiosis with a significant abundance of *Eggerthella lenta* and depletion of *Parabacteroides distasonis* and *Lactobacillus* spp., and increased the production of bile acid metabolites, especially taurodeoxycholic acid (TDCA), in the colon [153]. Bile acids are necessary for protecting the liver and other tissues from cholesterol toxicity, and they also regulate the composition of the GMB [154]. As a result of exposure to cigarette smoking, the accumulation of cholesterol (TC) in the liver increases due to the upregulated expression of cholesterol-synthesis-related genes. TC is converted into bile acids in the liver for excretion and re-absorbed in the intestine. This leads to the accumulation of bile acids in the gut, finally causing disturbance in the homeostasis of cholesterol and bile acid metabolism with GMB dysbiosis [155]. Furthermore, smoking tobacco cigarettes significantly decreased the abundance of *Bacteroides* and increased the abundance of *Prevotella* when compared to electronic cigarettes [156]. Tobacco smoke contains a variety of toxic gases, including carbon dioxide and CO. Each cigarette emits approximately 20–30 mL of CO, which converts approximately 15% of hemoglobin into carboxyhemoglobin, affecting O_2_ transport and resulting in systemic hypoxia [157]. Furthermore, hydrogen sulfide (H_2_S), which was considered the main toxic gas of tobacco smoke, was reported to alter the gut microbiota composition of weaning pigs by increasing the abundance of *Firmicutes* and *Proteobacteria*. In contrast, Bacteroides abundance decreased after 28 days of exposure to different levels of H_2_S [158].

## 10. Conclusions

The emerging connection between the gut microbiota and breast cancer paints a fascinating and complex picture. This review has highlighted various mechanisms by which these bacterial residents may influence disease initiation, progression, and treatment response. While much remains to be unraveled, the evidence is compelling enough to ignite optimism for novel breast cancer strategies. On the horizon, we should address intricate topics such as personalized microbiome profiling, targeted microbial manipulations, gut-based biomarkers, and synergy between conventional therapies and microbiome interventions, to open new gates for breast cancer therapy. However, significant challenges lie ahead. Large-scale, robust clinical trials are crucial for validating preclinical findings and establishing clear cause-and-effect relationships. Standardization of microbiome analysis techniques and improved understanding of individual microbial variations are also essential. Nevertheless, the potential of the gut microbiome in breast cancer is undeniable. By deciphering this intricate ecological landscape, we may tap into a potent approach to interventions, offering hope for a future where BC prevention and treatment are transformed.

## Data Availability

Data are included in the article.

## List of Abbreviations

AcH: Acetaldehyde; ADP: adenosine diphosphate; ALDH: Aldehyde dehydrogenase; AP-1: activator protein-1; AR: androgen receptor; BAK: BCL-2-antagonist/killer; BRCA 1: breast cancer gene 1; CAR: chimeric antigen receptor; CCL22: C–C motif chemokine 22; COX 2: cytochrome C oxidase, cyclooxidase; CYP27A1: cytochrome P 27A1; DDR: DNA damage repair; DHT: dihydrotestosterone; EGF: epidermal growth factor; EMT: epithelial and mesenchymal transition; ER: estrogen receptor; ERE: estrogen responsive element; ERK: extracellular-signal-regulated kinase; EVs: extracellular vesicles; FMT: fecal microbiota transplantation; FIH: factor-inhibiting HIF-1; GFR: growth factor receptor; GIT: gastrointestinal tract; Gli-1: glioma gene 1; GPR: G-protein-coupled receptor; GMB: gut microbiota; HDAC: histone deacetylase; HER: herceptin receptor; HIF-1 a: hypoxia-inducible factor 1-alpha; Hly-E: hemolysin-E; HRE: hypoxia-responsive element; IGF: insulin-like growth factor; IRF: interferon regulatory factor; LCA: lithocholic acid; LNCaP: androgen-sensitive human prostate adenocarcinoma cell line; LPS: lipopolysaccharide; MAPK: mitogen-activated protein kinase pathway; mTOR: mammalian target of rapamycin; NADPH: nicotinamide adenine dinucleotide phosphate; ncRNA: non-coding RNA; NFκB: nuclear factor kappa-light-chain-enhancer of activated B cells; NO: nitric oxide; NRF2: nuclear-factor-erythroid-2-related factor 2; PAHs: polycyclic aromatic hydrocarbons; PAMP: pathogen-associated molecular pattern; PI3K/AKT/mTOR: PI3K: phosphatidylinositol 3-kinase; AKT: protein kinase B; mTOR: mammalian target of rapamycin; PPI: Keap1-Nrf2 protein–protein interaction; PR: progesterone receptor; PHDs: prolyl-hydroxylases; Ras/Raf/MEK/ERK: signal transduction pathway; Ras: renin–angiotensin system protein; Raf: rapidly accelerated fibrosarcoma; MEK: mitogen-activated protein kinase; ROS: reactive oxygen species; SAGE: serial analysis of gene expression; SCFAs: short-chain fatty acids; SGL: spore of *Ganoderma lucidum*; SHH: Sonic Hedgehog-Gli1 pathway; SN-38G: metabolite SN-38 glucuronide; Sp1: proximal specificity protein 1; TDCA: taurodeoxycholic acid; TGR5: Takeda G protein-coupled receptor 5; TLR: Toll-like receptor; TMAO: trimethylamine N-oxide; TME: tumor microenvironment; TNBC: triple-negative breast cancer.
